# Supplementary material for: Deletions in Glial Fibrillary Acidic Protein Leading to Alterations in Intermediate Filament Assembly and Network Formation
Source: Int J Mol Sci. 2025 Feb 23;26(5):1913. doi: 10.3390/ijms26051913 (PMC11900225; doi:10.3390/ijms26051913)
Supplement: Supplementary file 1 [file ijms-26-01913-s001.zip › ijms-3387539-supplementary.pdf]

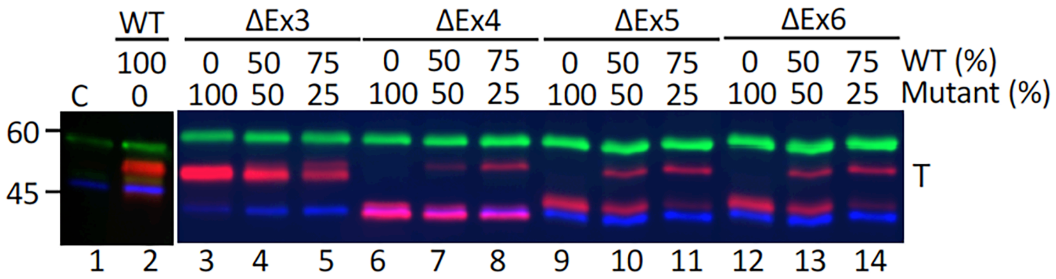

Figure S1. Expression levels of GFAP in transduced astrocytes. Primary astrocytes derived from GFAP-null rats were either untransduced (lane 1) or transduced with indicated GFAP expression constructs (2-14), either alone or in combination with indicated proportions of WT GFAP. At 72 hours after transduction, cells were extracted and the total cell lysates were analyzed by immunoblotting using a polyclonal anti-panGFAP, anti-actin (blue channel) and anti-vimentin (green channel) antibodies.
